# Quantitative Analysis of Parkinsonian Tremor in a Clinical Setting Using Inertial Measurement Units

**DOI:** 10.1155/2018/1683831

**Published:** 2018-06-21

**Authors:** Donatas Lukšys, Gintaras Jonaitis, Julius Griškevičius

**Affiliations:** Faculty of Mechanics, Department of Biomechanical Engineering, Vilnius Gediminas Technical University, Basanavičiaus str. 28, LT-03224 Vilnius, Lithuania

## Abstract

**Background:**

Parkinson's disease (PD) is a neurodegenerative disorder that affects human voluntary movements. Tremor is one of the most common symptoms of PD and is expressed as involuntary oscillation of the body. Tremors can be analysed in the frequency domain.

**Objective:**

The aim of the current study was to examine selected tremor parameters (frequency, root mean square, and approximated entropy) in order to quantify the characteristics of patients diagnosed with PD, compared to a healthy control group, and to compare the parameters by dividing the subjects according to UPDRS assessment.

**Methods:**

The subjects were divided into two groups: a group of people diagnosed with PD (*n* = 19) and a control group consisting of healthy volunteers (CO = 12). Each subject performed motor tasks specific to certain tremors: the finger-to-nose test. Each subject performed a motor task three times. A nine degree of freedom (DOF) wireless inertial measurement unit was used for the measurement of upper limb motor tasks. For the quantitative estimation of kinetic and postural tremors, dominant frequency, root means square, and approximation entropy were selected and calculated from the measured angular velocity and linear acceleration signals. A one-way ANOVA with a significance level of *α* = 0.05 was used to test the null hypothesis that the means of the tremor metrics were the same between the PD and CO groups.

**Results:**

Statistically significant differences between PD patients and control groups were observed in ApEn acceleration signal of kinetic tremor, ApEn angular velocity signal of kinetic tremor, ApEn angular velocity of postural tremor, frequency acceleration signal of postural tremor, and RMS angular speed kinetic tremor.

**Conclusion:**

Application of inertial measurement units for clinical research of patients and PD tremor evaluation allows providing quantitative information for diagnostic purposes, during screening in a clinical setting that differentiates between PD patients and controls.

## 1. Introduction

Parkinson's disease (PD) is a disorder of certain nerve cells in the part of the brain that produces dopamine. PD usually begins in middle or later life (after age 50) [1]. PD is the second most common neurodegenerative movement disorder [2]. Tremor, in addition to rigidity, bradykinesia, and postural instability, is generally considered to be one of the cardinal features of PD [3].

Tremor is defined as rhythmical and involuntary oscillatory movement of a body part; detection of tremors plays a crucial role in the management and treatment of PD patients. There are three types of PD tremor:

1. rest tremor, which occurs in a body segment while this body segment is relaxed;
2. action (kinetic) tremor, which is associated with any voluntary movement;
3. postural tremor, which occurs when a person maintains a position against gravity, such as holding their arms outstretched.

Postural or action tremors can happen together with rest tremors, but with different frequencies. A rest tremor can occur with a postural tremor, but disappears during an action tremor task [4].

The Unified Parkinson's Disease Rating Scale (UPDRS) allows evaluation of motor and nonmotor symptoms in PD. This scale has standardised movements and tasks; thus, doctors do not need to use any special devices to evaluate specific movements. The severity of the disease is evaluated only according to the competence of the doctor. Each motor task is rated from 0 to 4, where 0 is normal and 4 is severe.

Various motion capture equipment can be used to quantify tremors. An accelerometer is one of the most commonly used sensors for tremor detection [5]. Electromyography (EMG) is also used to detect tremors of the limbs [6], and laser displacement sensors can be used to measure and quantify tremors [7]. There are also systems that allow the registration of tremors and the parameters associated with disease rating scales [8]. Inertial measurement units (IMUs) are increasingly used to detect tremors, as they combine several types of sensors: accelerometers, gyroscopes, and magnetometers. In some studies, angular velocity is used to quantify tremors instead of the acceleration signal [9].

PD can be diagnosed incorrectly and can be confused with other diseases such as essential tremor (ET) [10]. Therefore, studies use data classification techniques to discriminate different diseases. A support vector machine has been used successfully to classify PD and ET tremor characteristics [11]. Classical statistical techniques such as binary logistic linear regression and linear discriminant analysis can also be applied [12]. Researchers often use artificial neural network methods which can automatically detect PD resting tremor using EMG and a recurrent neural network classifier [13].

Tremor signal analysis can be divided into several types: time-domain analysis [14], spectral analysis [15], time-frequency analysis [16], and nonlinear analysis [17].

Amplitude and frequency are the main parameters that describe a tremor [18]. These parameters allow researchers to distinguish between different types of tremors and to assess the severity of the disease. Fast Fourier transformation is used to obtain frequency characteristics. This is a mathematical technique for transforming a signal from the time domain to the frequency domain. Most research analyses only a single limb, usually the one that is most affected, or analyses one segment of the upper limb. Further studies often use a combination of parameters in order to separate PD patients from the control group. In addition, quantification of tremors is often performed using a dominant frequency, which is calculated using the power spectral density function or root mean square (RMS). One of the most commonly used nonlinear analysis parameters is the approximated entropy, which allows estimation of the complexity of the signal.

The aim of the current study was to examine selected parameters (dominant frequency (*f*), root mean square (RMS), and approximated entropy (ApEn)) in order to quantify the characteristics of patients with PD, compared to a control group, and to compare the parameters by dividing the subjects according to UPDRS assessment (kinetic or postural tremor). Finally, this study aimed to compare which side and segment (s) were most affected.

## 2. Materials and Methods

The data were collected at the Vilnius University Hospital “Santaros Klinikos” Centre of Neurology. Subjects were divided into two groups: a group of subjects diagnosed with PD and healthy subjects. The control subjects did not have any illnesses and injuries that would impair movement or coordination. The inclusion criterions were person older than 18 years of age, able to walk independently without assisting devices, and disease severity, according to the Hoehn and Yahr scale, at 2-3. The exclusion criterions were cardiologic pathologies and other diseases that would impair movement. The experimental protocol was approved by the local ethical committee and all the subjects gave their written informed consent before participating. Subject data are presented in Table 1.

**Figure 1:**
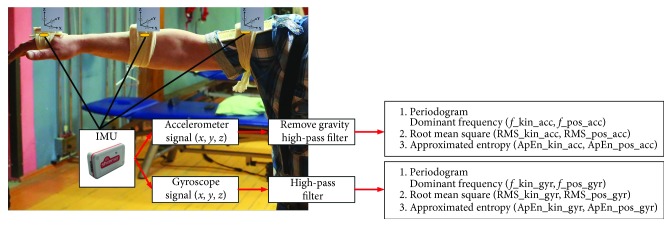
Placement of the inertial measurement unit (IMU) sensors on the upper extremity and the calculation algorithm.

A nine degree of freedom (DOF) wireless inertial measurement unit (Shimmer Research, Dublin, Ireland) was used for the measurement of upper limb motor tasks. Six wireless sensors were attached to the subjects' right and left arm, forearm, and hand (Figure 1).

Each sensor measured linear acceleration (three-axis acceleration, FreeScale MM7361, and accelerometer limit ± 6 g), angular velocity (three-axis gyroscope, InvenSense 500 MEMs Gyro, angular velocity limit ± 500°/s, and sensibility 2 mV/°), and magnetic heading (three-axis magnetometer, Honeywell HMX5843, and input field boundaries = −0.7–4.5 Ga). The data from the sensors were received via a Bluetooth wireless connection, at a sampling frequency (*F*_s_) of 51.2 Hz, and were stored on a computer.

Each subject performed motor tasks specific to certain tremors: the finger-to-nose test for examining kinetic tremor features and holding an outstretched arm for examination of postural tremor features. Each subject performed a motor task three times.

**Figure 2:**
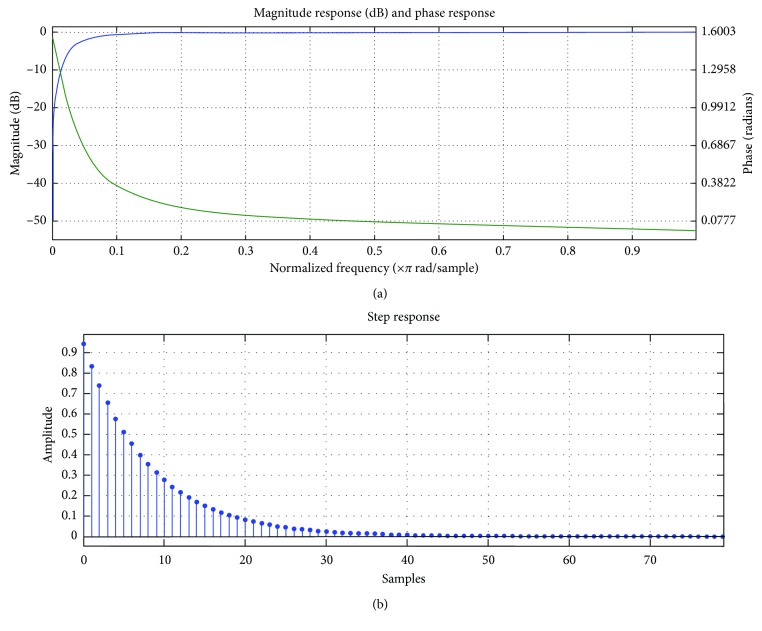
(a) Frequency-domain digital filter; (b) time-domain digital filter.

Data processing was performed using Matlab (MathWorks, Inc., 2013). Prior to the analysis, all data recordings were high-pass filtered with a cut-off frequency of 1 Hz (1st order Butterworth filter). The cut-off frequency was chosen considering the digital signal processing to the IMU. Further, the gravitational component was removed from the acceleration signal.  Figure 2 shows time and frequency response of a digital filter.

For the quantitative estimation of kinetic and postural tremors, several parameters were selected and calculated from the measured angular velocity and linear acceleration signals:

- Dominant frequency (frequency kinetic tremor acceleration signal (*f*__kin_acc_), frequency postural tremor acceleration signal (*f*__pos_acc_), frequency kinetic tremor gyroscope signal (*f*__kin_gyr_), and frequency postural tremor gyroscope signal (*f*__pos_gyr_))
- Root mean square (RMS kinetic tremor acceleration signal (RMS__kin_acc_), RMS postural tremor acceleration signal (RMS__pos_acc_), RMS kinetic tremor gyroscope signal (RMS__kin_gyr_), and RMS postural tremor gyroscope signal (RMS__pos_gyr_))
- Approximated entropy (ApEn kinetic tremor acceleration signal (ApEn__kin_acc_), ApEn postural tremor acceleration signal (ApEn__pos_acc_), ApEn kinetic tremor gyroscope signal (ApEn__kin_gyr_), and ApEn postural tremor gyroscope signal (ApEn__pos_gyr_)).

Spectral analysis was performed to identify dominant frequencies. The signal strength in a specific frequency spectrum is shown using power spectral density (PSD). The dominant frequency of a tremor is evident as a visible peak in the PSD [19]. A periodogram was used for the evaluation of PSD. A periodogram is a nonparametric estimate of power spectral density, which is based on the Fourier transform of the based estimate of the autocorrelation sequence. A rectangular window is used for calculating the PSD. A periodogram is defined as

(1) $$\begin{array}{c} P_{xx}\left(f\right)=\frac{1}{LF_{S}}{\left|\overset{L-1}{\underset{n=0}{\sum }}x_{L}\left(n\right)e^{-i2\pi fn/F_{s}}\right|}^{2}, \end{array}$$

where *x*_*L*_(*n*) is the signal, *L* is the length, and *F*_S_ is the sampling frequencies.

If the dominant frequency in different axes is not the same, the valid dominant frequency in the axis with the highest peak power is regarded as the dominant frequency of all axes.

Approximate entropy (ApEn) is a technique that quantifies the degree of irregularity and the unpredictability of fluctuations in time series data [20]. This is a popular tool for analysing the complexity of time series data, especially in clinical research. Low ApEn values indicate predictability and high regularity of time series data, whereas high ApEn values indicate incalculable and random time series data. In this study, we calculated ApEn values for all data sets using *m* = 2 and *r* = 0.45 of SD of the individual subjects' time series. This value is recommended [21], and ApEn is defined as

(2) $$\begin{array}{c} \text{ApEn}\left(S_{n},m,r\right)=\ln\left[\frac{c_{m}\left(r\right)}{c_{m+1}\left(r\right)}\right], \end{array}$$

where *S*_*n*_ gives a sequence consisting of *N* instantaneous measurements, *m* specifies the pattern length, *r* defines the criterion of similarity, and *C*_im_(*r*) is the fraction of patterns of length *m* that is similar to the pattern of the same length that starts at interval *i*.

RMS is used to evaluate intensity of tremors. RMS interprets actual vibration levels, while PSD results indicate the dominant frequency that contributes the most to the tremor. Because tremors are based on a dominant frequency, the advantages of PSD compared to a statistical measure (quadratic mean) are that it isolates the tremor signals from noise and other movements, by analysis of the frequency dimension, and it provides a squared value for the signals.

Data from PD patients were divided into groups according to a clinical assessment: Right 0, Right 1, Left 0, and Left 1. Statistical analysis of the metrics was performed using IBM's SPSS v22 software. A one-way ANOVA with a significance level of *α* = 0.05 was used to test the null hypothesis that the means of the tremor metrics were the same between the PD and CO groups.

## 3. Results

Significant differences (*α* < 0.05) between the PD and CO groups are shown in Tables 2–4 (bold values have statistical significance).

**Figure 3:**
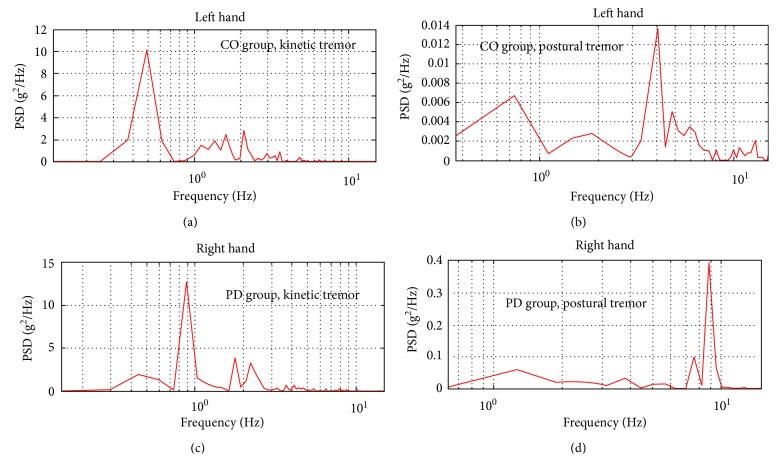
The power spectrum based power spectral density (PSD) plot. (a, b) Postural and kinetic tremor in the CO group; (c, d) kinetic and postural tremor in the PD group.

Data from PD patients were further divided into groups according UPDRS clinical assessment (action or postural tremor of hand and UPDRS III motor task 21). This motor task is evaluated in numbers from 0 to 4 (0: no tremor; 4: severe tremor). This motor task is assessed by the right and left side of subjects. This assessment was received from a doctor. Clinical assessment was performed, and the upper limbs from both sides were evaluated; each segment was then scored (upper arm, forearm arm, and hand). Each participant was classified according to the clinical assessment (Right 0 (*n* = 14), Right 1 (*n* = 4), Left 0 (*n* = 14), and Left 1 (*n* = 4)). The data were grouped as follows: Left 0 versus Left 1 and Right 0 versus Right 1.  Figure 3 shows the PSD calculation from the acceleration signal.

Statistically significant differences between the PD groups with regard to the UPDRS assessment are shown in Table 5 (bold values have statistical significance).

## 4. Discussion

Application of inertial measurement units for clinical research of patients and PD tremor evaluation allows providing quantitative information for diagnostic purposes, during screening in a clinical setting that differentiates between PD patients and controls. Three basic parameters (frequency, RMS, and approximation entropy) can be used to separate two different groups and for quantitative tremor assessment according to the UPDRS score.

As can be seen from the results obtained (Tables 2–4) to find statistically significant differences between the calculated parameters between PD and CO groups, ApEn is the best way to separate the groups from each other. The result obtained for ApEn values is higher in the PD group, which indicates that the movement is more unpredictable and more incidental. Higher values of ApEn between the subjects and the different sides indicate which side and segment are more severe in the PD group. RMS values show tremor intensity. The result shows that the intensity of the postural tremor is larger on the left side of the angular velocity signal. The dominant frequency is one of the main characteristics for estimating PD tremor, and the obtained result indicates that postural tremor detection is a more appropriate angular velocity signal than the acceleration signal.

Divided PD patients according UDPRS clinical assessment showed a statistically difference. The higher values of the calculated parameter indicate that the values set by the medical doctor correspond to the difference between the calculated values, and their values are higher among the estimates.

## Figures and Tables

**Table 1:** Demographic and clinical characteristic of subjects.

Group	*n*	Total UDPRS score (mean ± SD)	UPDRS III score (mean ± SD)	Age (mean ± SD)	Hoehn and Yahr scale score (mean ± SD)
PD	19 (M/F: 8/11)	40.21 ± 15.97	28.42 ± 11.21	61.53 ± 10.81	2.10 ± 0.54
CO	12 (M/F: 6/6)	—	—	57.83 ± 7.58	—

M, male; F, female.

**Table 2:** Comparison of approximation entropy between PD and CO.

Segment	Group	ApEn__kin_acc_ (mean ± SD)	ApEn__pos_acc_ (mean ± SD)	ApEn__kin_gyr_ (mean ± SD)	ApEn__pos_gyr_ (mean ± SD)
Right upper arm	CO	0.452 ± 0.139	0.967 ± 0.168	0.394 ± 0.107	0.455 ± 0.188
	PD	0.578 ± 0.208	0.947 ± 0.154	0.416 ± 0.175	0.552 ± 0.135

Left upper arm	CO	**0.368 ± 0.195**	0.991 ± 0.153	**0.329 ± 0.097**	0.541 ± 0.223
	PD	**0.552 ± 0.198**	0.860 ± 0.181	**0.475 ± 0.119**	0.577 ± 0.168

Right forearm	CO	**0.240 ± 0.081**	0.859 ± 0.201	0.383 ± 0.178	**0.429 ± 0.151**
	PD	**0.387 ± 0.194**	0.906 ± 0.217	0.375 ± 0.098	**0.573 ± 0.197**

Left forearm	CO	0.297 ± 0.115	0.891 ± 0.229	**0.279 ± 0.069**	0.516 ± 0.183
	PD	0.491 ± 0.227	0.858 ± 0.281	**0.369 ± 0.104**	0.579 ± 0.200

Right hand	CO	0.343 ± 0.112	0.776 ± 0.156	0.283 ± 0.070	0.402 ± 0.124
	PD	0.400 ± 0.181	0.762 ± 0.210	0.311 ± 0.139	0.494 ± 0.179

Left hand	CO	0.373 ± 0.111	0.804 ± 0.180	0.291 ± 0.095	0.463 ± 0.180
	PD	0.447 ± 0.155	0.754 ± 0.194	0.321 ± 0.103	0.482 ± 0.169

**Table 3:** Comparison of root mean square between PD and CO.

Segment	Group	RMS__kin_acc_ (mean ± SD)	RMS__pos_acc_ (mean ± SD)	RMS__kin_gyr_ (mean ± SD)	RMS__pos_gyr_ (mean ± SD)
Right upper arm	CO	1.617 ± 0.958	0.275 ± 0.095	34.078 ± 19.754	0.394 ± 0.107
	PD	1.629 ± 1.007	0.267 ± 0.159	36.018 ± 21.575	0.416 ± 0.175

Left upper arm	CO	2.349 ± 1.595	0.256 ± 0.130	43.031 ± 20.821	**0.329 ± 0.097**
	PD	1.576 ± 1.008	0.317 ± 0.289	31.536 ± 16.057	**0.475 ± 0.119**

Right forearm	CO	1.909 ± 1.006	0.344 ± 0.198	45.722 ± 23.602	0.383 ± 0.178
	PD	1.772 ± 0.882	0.281 ± 0.157	42.203 ± 16.655	0.375 ± 0.098

Left forearm	CO	1.834 ± 0.831	0.271 ± 0.215	63.640 ± 25.549	**0.279 ± 0.069**
	PD	1.469 ± 0.794	0.239 ± 0.101	44.170 ± 16.903	**0.369 ± 0.104**

Right hand	CO	2.429 ± 1.350	0.392 ± 0.204	70.972 ± 39.044	0.283 ± 0.070
	PD	2.703 ± 1.587	0.395 ± 0.209	86.217 ± 48.488	0.310 ± 0.139

Left hand	CO	2.446 ± 1.588	0.377 ± 0.283	70.419 ± 35.286	0.290 ± 0.095
	PD	2.462 ± 1.230	0.476 ± 0.406	88.970 ± 44.162	0.321 ± 0.103

As can be seen, selected parameters (ApEn, dominant frequency, and RMS) allowed us to distinguish between PD and CO groups. Therefore, these parameters allow quantification of tremor characteristics.

**Table 4:** Comparison of dominant frequencies between PD and CO.

Segment	Group	*f*__kin_acc_ (mean ± SD)	*f*__pos_acc_ (mean ± SD)	*f*__kin_gyr_ (mean ± SD)	*f*__pos_gyr_ (mean ± SD)
Right upper arm	CO	3.278 ± 1.565	6.558 ± 3.213	2.175 ± 0.752	4.164 ± 2.162
	PD	3.516 ± 2.094	7.610 ± 2.730	2.519 ± 1.075	5.715 ± 2.836

Left upper arm	CO	3.101 ± 1.374	5.788 ± 2.775	2.007 ± 0.866	**3.195 ± 1.633**
	PD	3.925 ± 1.882	7.588 ± 3.627	2.814 ± 1.377	**5.421 ± 2.081**

Right forearm	CO	1.534 ± 0.396	**3.196 ± 1.949**	1.829 ± 0.623	2.693 ± 1.662
	PD	1.878 ± 0.890	**5.234 ± 2.947**	2.364 ± 1.405	4.248 ± 2.654

Left forearm	CO	1.944 ± 0.827	3.958 ± 3.373	2.079 ± 0.749	**2.639 ± 1.834**
	PD	2.173 ± 1.112	6.617 ± 4.504	2.722 ± 2.155	**5.074 ± 2.841**

Right hand	CO	1.750 ± 0.415	2.287 ± 1.771	1.296 ± 0.649	2.316 ± 2.062
	PD	1.653 ± 0.488	3.857 ± 2.682	1.037 ± 0.347	3.484 ± 2.400

Left hand	CO	1.939 ± 0.656	4.009 ± 3.151	1.548 ± 0.723	2.396 ± 1.211
	PD	1.882 ± 1.014	2.892 ± 1.889	1.662 ± 1.726	3.333 ± 2.479

**Table 5:** Tremor parameters between the PD groups with regard to the UPDRS assessment.

Parameter	Segment	Right 0 (mean ± SD)	Right 1 (mean ± SD)	Segment	Left 0 (mean ± SD)	Left 1 (mean ± SD)
*f*__kin_gyr_	Right upper arm	2.486 ± 0.891	3.077 ± 1.423	Left upper arm	2.804 ± 1.303	3.335 ± 1.551
	Right forearm	2.082 ± 1.189	2.842 ± 1.915	Left forearm	2.354 ± 1.388	2.756 ± 3.245
	Right hand	1.063 ± 0.378	0.890 ± 0.236	Left hand	1.160 ± 0.363	1.954 ± 2.140

*f*__kin_acc_	Right upper arm	3.800 ± 2.234	3.177 ± 1.361	Left upper arm	3.512 ± 1.514	4.146 ± 1.568
	Right forearm	1.940 ± 0.982	1.759 ± 0.693	Left forearm	2.561 ± 0.914	1.232 ± 0.994
	Right hand	1.709 ± 0.528	1.585 ± 0.336	Left hand	2.103 ± 1.101	1.277 ± 0.198

*f*__post_gyr_	Right upper arm	5.730 ± 2.901	6.835 ± 1.654	Left upper arm	5.111 ± 1.802	7.224 ± 2.090
	Right forearm	3.861 ± 2.598	6.342 ± 1.882	Left forearm	5.103 ± 2.724	6.101 ± 2.843
	Right hand	2.927 ± 2.534	5.373 ± 0.708	Left hand	2.692 ± 2.177	5.238 ± 2.99

*f*__post_acc_	Right upper arm	7.263 ± 3.065	8.476 ± 1.338	Left upper arm	7.210 ± 3.962	9.109 ± 2.638
	Right forearm	4.642 ± 3.149	6.960 ± 1.711	Left forearm	6.883 ± 5.155	5.987 ± 2.281
	Right hand	**2.881 ± 2.2821**	**6.463 ± 1.884**	Left hand	**2.189 ± 1.5860**	**4.716 ± 1.281**

RMS__kin_gyr_	Right upper arm	37.44 ± 21.235	38.85 ± 22.193	Left upper arm	33.69 ± 13.926	29.344 ± 22.912
	Right forearm	**45.37 ± 16.075**	**38.445 ± 13.822**	Left forearm	**48.706 ± 15.478**	**34.746 ± 15.483**
	Right hand	94.38 ± 50.830	75.52 ± 27.201	Left hand	**102.949 ± 41.806**	**53.629 ± 23.809**

RMS__kin_acc_	Right upper arm	1.5377 ± 0.684	2.237 ± 1.755	Left upper arm	1.741 ± 0.981	1.301 ± 1.100
	Right forearm	1.8329 ± 0.628	1.912 ± 1.500	Left forearm	1.641 ± 0.803	1.024 ± 0.677
	Right hand	2.6642 ± 1.335	3.395 ± 2.262	Left hand	2.729 ± 1.107	2.017 ± 1.382

RMS__post_gyr_	Right upper arm	4.8751 ± 3.650	5.218 ± 3.298	Left upper arm	3.974 ± 2.542	4.452 ± 2.172
	Right forearm	5.1201 ± 3.841	5.870 ± 1.942	Left forearm	3.983 ± 2.160	4.518 ± 1.867
	Right hand	8.261 ± 7.465	6.589 ± 1.707	Left hand	8.774 ± 7.580	7.808 ± 6.445

RMS__post_acc_	Right upper arm	0.238 ± 0.087	0.389 ± 0.303	Left upper arm	0.296 ± 0.209	0.411 ± 0.546
	Right forearm	**0.292 ± 0.178**	**0.259 ± 0.073**	Left forearm	**0.249 ± 0.110**	**0.203 ± 0.078**
	Right hand	0.423 ± 0.212	0.351 ± 0.210	Left hand	0.504 ± 0.436	0.438 ± 0.376

ApEn__kin_gyr_	Right upper arm	0.381 ± 0.134	0.514 ± 0.291	Left upper arm	**0.443 ± 0.108**	**0.585 ± 0.116**
	Right forearm	**0.364 ± 0.097**	**0.407 ± 0.123**	Left forearm	**0.351 ± 0.114**	**0.434 ± 0.041**
	Right hand	0.307 ± 0.144	0.283 ± 0.130	Left hand	0.305 ± 0.109	0.375 ± 0.087

ApEn__kin_acc_	Right upper arm	0.556 ± 0.196	0.561 ± 0.210	Left upper arm	0.513 ± 0.167	0.600 ± 0.252
	Right forearm	**0.333 ± 0.152**	**0.462 ± 0.190**	Left forearm	**0.445 ± 0.183**	**0.593 ± 0.352**
	Right hand	0.389 ± 0.181	0.353 ± 0.133	Left hand	0.405 ± 0.116	0.522 ± 0.207

ApEn__post_gyr_	Right upper arm	0.564 ± 0.152	0.542 ± 0.056	Left upper arm	0.579 ± 0.187	0.579 ± 0.138
	Right forearm	**0.570 ± 0.186**	**0.645 ± 0.233**	Left forearm	**0.549 ± 0.201**	**0.678 ± 0.216**
	Right hand	0.476 ± 0.179	0.532 ± 0.210	Left hand	0.445 ± 0.166	0.595 ± 0.162

ApEn__post_acc_	Right upper arm	0.970 ± 0.129	0.845 ± 0.227	Left upper arm	0.879 ± 0.177	0.803 ± 0.232
	Right forearm	**0.898 ± 0.201**	**0.698 ± 0.309**	Left forearm	**0.867** ± **0.273**	**0.678 ± 0.386**
	Right hand	0.738 ± 0.199	0.792 ± 0.273	Left hand	0.750 ± 0.144	0.745 ± 0.364

## Data Availability

The data used to support the findings of this study are available from the corresponding author upon request.
